# Thirteen Cases of Pulmonary Pneumocystis in HIV-Negative Patients

**DOI:** 10.7759/cureus.43409

**Published:** 2023-08-13

**Authors:** Abir Bouhamdi, Btissame Es-sabbahi, Rajae Amiali, Mounia Serraj, Mohamed Biaz, Mohamed Chakib Benjelloun, Bouchra Amara

**Affiliations:** 1 Pulmonology Department, Hassan II University Hospital, University of Sidi Mohammed Ben Abdellah, Fez, MAR

**Keywords:** pneumocystis jirovecii, prophylaxis, induced sputum, lymphopenia, immunosupression

## Abstract

We report 13 cases of pulmonary pneumocystis (PCP) in human immunodeficiency virus (HIV)-uninfected patients. Of eight males and five females, with a mean age of 55 years, one had breast neoplasia, two had common variable immunodeficiency (CVID), one had an autoimmune disease "Goodpasture's syndrome", and one had idiopathic fibrosis (nonspecific interstitial pneumonia/fibrosis (NIP)) undergoing prolonged corticosteroid therapy for two years, with no known immunosuppression in the remaining cases. The clinical picture was characterized by constant dyspnea and severe hypoxia in 11 cases. Lymphopenia was present in nine cases with an average rate of 920.76 elements/mm^3^. The diagnosis was confirmed by isolation of *Pneumocystis jirovecii* (PCJ) from induced sputum, except in two cases where analysis of bronchoalveolar lavage (BAL) fluid was required. With trimethoprim/sulfamethoxazole (TMP/SMX) and corticosteroid therapy, the course was favorable in all cases. Prophylactic treatment was indicated in three cases.

## Introduction

*Pneumocystis jirovecii* (PCJ), a ubiquitous fungus, is the cause of the serious opportunistic disease known as pulmonary pneumocystis (PCP), which affects immunocompromised individuals [1]. Described as a severe disease in the early 1960s, it was nevertheless considered rare until the onset of the acquired immunodeficiency syndrome (AIDS) epidemic in 1982.

Its prognosis remains poor, and it appears to be on the increase in patients who are seronegative for the human immunodeficiency virus (HIV), solid organ transplant recipients, patients treated for hematologic malignancies (mainly lymphoproliferative), patients with autoimmune or inflammatory diseases, and those receiving long-term corticosteroids. These pneumonias are associated with higher morbidity and mortality than AIDS (30%-60% versus 10% for HIV-infected patients), hence the need for early detection and treatment [2].

The aim of our study is to analyze the clinical and paraclinical characteristics of people with PCP and identify the immunodepressions (other than HIV) most at risk for this infection.

## Materials and methods

Study design

This is a two-year retrospective study (January 1, 2021, to December 31, 2022) of patients with pulmonary pneumocystis. The study was carried out in the Pneumology Department of Hassan II University Hospital in Fez.

Study population

Inclusion Criteria

The patients included are subjects aged 15 years and over, of all sexes, recruited from the Pneumology Department of Fez University Hospital over a two-year period (January 1, 2021, to December 31, 2022). Only HIV-negative patients were included, with HIV serology performed in all PCP cases.

Exclusion Criteria

We excluded HIV-positive patients.

Data collection

Data were collected from patients' computerized medical records. Analysis of clinical records included epidemiological data (age, sex, and date of onset), history (in particular immunosuppressive disease), and immunosuppressive treatments received. The clinical, biological, radiological, and microbiological data of the PCP episode were collected, as well as the treatments received and their evolution.

Statistical analysis

Data entry was performed using Microsoft Excel (Microsoft Corp., Redmond, WA, USA), and analysis was performed using Statistical Package for the Social Sciences (SPSS) version 25 (IBM SPSS Statistics, Armonk, NY, USA) at the epidemiology laboratory of the Faculty of Medicine and Pharmacy of Fez, Sidi Mohamed Ben Abdellah University.

Ethical considerations

All patients who participated in the study gave their consent, and data were collected anonymously. Data confidentiality was ensured during data collection and processing.

## Results

A total of 13 cases were gathered, with a sex ratio of eight males to five females (1.6) and an average age of 55.23±15.44 (28-75 years). The median age was 59 years. A third of patients (30.76%) reported actively smoking.

The underlying diseases responsible for immunosuppression were as follows: one case had Goodpasture's syndrome with end-stage renal failure on chronic hemodialysis, having received five boluses of cyclophosphamide followed by azathioprine; two patients had a common variable immunodeficiency (CVID); one patient had carcinoma of the left breast and had been receiving chemotherapy for six months; and the fifth patient had idiopathic fibrosis (nonspecific interstitial pneumonia/fibrosis (NIP)) on long-term corticosteroid therapy that started two years previously, combined with azathioprine for one year.

The median period from the onset of symptoms to the diagnosis of PCP was 28 days, with a range of 5-62 days. In five cases, the beginning was gradual. In eight cases, it was sudden, with an average time between the onset of symptoms and diagnosis of 17 days.

Clinically, all of the patients were dyspneic at the time of management, with 11 of them experiencing respiratory distress. Of the patients, 85% also had productive coughs, and 77% of them were feverish.

In terms of biology, the mean circulating lymphocyte count was 920.76 cells/mm^3^, with a median of 640 cells/mm^3^. In any case, CD4+ cells were not counted. The C-reactive protein level was 132 mg/L.

**Figure 1:**
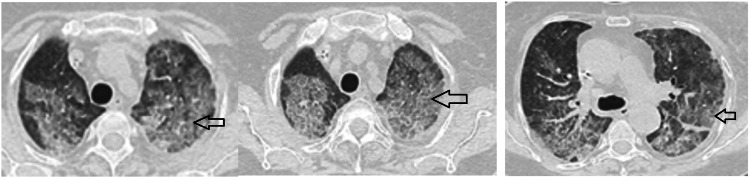
A 67-year-old female patient's CT scan reveals diffuse ground-glass regions through the center and periphery of both lung hemifields Arrows: ground-glass opacities CT: computed tomography

On thoracic computed tomography (CT), 10 patients had diffuse bilateral pneumopathy with ground-glass areas in six cases and condensation foci in four others. Two cases had an atypical appearance with pleural collections, and one case had sequelae of bronchial dilatation (Figure 1).

With the exception of two cases where examination of the bronchoalveolar lavage (BAL) fluid was required, all diagnoses were based on the isolation of *Pneumocystis jirovecii* in the induced sputum. The cytobacteriological examination of sputum (ECBC) and BAL fluid analyses revealed no coinfections in any patients. An average of 29 days passed between the onset of symptoms and the beginning of treatment.

Trimethoprim/sulfamethoxazole (TMP/SMX) was administered orally to all individuals. One instance of cotrimoxazole intolerance was noted with the emergence of a fixed erythema pigmentosa, which prompted the drug's withdrawal to try tolerance induction before suggesting an alternative in the event of failure.

Corticosteroid medication was given to eight (62%) individuals who had PCP. The mean room air arterial oxygen saturation (SpO2) for patients who received corticosteroid therapy was 65.62%. Five patients needed oxygen therapy for an extra month, one patient needed noninvasive ventilation for his underlying chronic lung condition, and four patients needed noninvasive ventilation. All of these patients were alive after being hospitalized. There were no reported fatalities.

Eighteen days on average were spent in the hospital. Eight days was the shortest time frame, and 51 days was the longest time frame.

Three cases received a prophylactic dose of TMP/SMX: one was receiving cyclophosphamide and long-term corticosteroid therapy for his anti-synthetase syndrome, which was identified concurrently with his PCJ infection; another was receiving long-term azathioprine and chronic hemodialysis; and the third case had a common variable immunodeficiency with PCJ colonization.

## Discussion

In the past, pulmonary pneumocystis was a rare pathology that exclusively affected kids with acute lymphocytic leukemia, severe malnutrition, and congenital cellular immunity abnormalities and organ transplant patients [3,4].

In the 1980s, when acquired immunodeficiency syndrome (AIDS) emerged, pneumocystis was observed in 60%-80% of HIV-positive patients [5]. With advances in prophylaxis and antiretroviral treatment, the number of cases fell considerably, reaching a value of 0.3 cases/100 people/year in March 1998.

The bulk of pneumocystis cases currently reported in developed nations include non-HIV-infected patients who fall into other risk groups [6,7].

*Pneumocystis jirovecii* is a ubiquitous fungus specific to the human species that is responsible for a primary infection most often asymptomatic in children [8]. The corresponding antibodies are detected as early as four years of age [9].

The prevalence of PCP appears to be rising in patients without HIV infection [5,10], especially in those with hematologic malignancies, those receiving chemotherapy for cancer [5], those who have had a solid organ or marrow transplant, and anyone else receiving corticosteroid and/or immunosuppressive therapy. PCP appears to be caused more by Wegener's granulomatosis than other dysimmune illnesses. Two of the patients in our series had common variable immunodeficiency (CVID), one had breast cancer, one had an autoimmune condition (Goodpasture's syndrome), and another was receiving prolonged corticosteroid therapy.

Between the fourth and fifth decades, pulmonary pneumocystis occurs often in HIV-negative people. The average age of the 28 patients in a study by Ficko et al. [4,11] was 52.8 years, the three instances in a study by Battikh et al. [10] had an average age of 50 years, and the average age of our series was 55 years, with no clear sex preponderance (Table 1). It is distinguished by a more abrupt onset with an average delay of 5-6 days [6,10], by more frequent and intense dyspnea (occurring in about 87% of cases in the literature), by more severe hypoxia necessitating more frequently mechanical breathing during this illness (in 60%-70% of cases), and by other characteristics [4,12,13]. The literature is therefore supported by our findings.

**Table 1:** Immunosuppressive illness frequency in PCP patients without HIV PCP: pulmonary pneumocystis, HIV: human immunodeficiency virus

Study	Hematologic cancer	Vasculitis systemic	Immune disorder	Cirrhosis	Organ transplantation	Solid cancer	Long-term corticosteroids	Immunosuppressant or biotherapy	Chemotherapy	Mortality
Ficko et al. (n=28) [11]	9 (marrow transplants)	2	4	1			10 (at the time of diagnosis), 23 (history of corticosteroids)	10 (at the time of diagnosis), 10 (history of immunosuppressants), 5 biotherapies	1 (at the time of diagnosis), 5 (history of chemotherapy)	7
Roblot et al. (n=103) [14]	18	27			24	18	57	22		
Battikh et al. (n=3) [10]	1 (lymphoma)	1 (Wegener's granulomatosis)				1 (breast neoplasia)		3 (at the time of diagnosis)	2	2
Our series (n=12)	-	-	1 (Goodpasture's syndrome)	-	-	1 (breast neoplasia)	1	3	1	0

The median number of circulating lymphocytes in HIV-uninfected immunocompromised patients is 500/mm^3^ (278-880/mm^3^). The median in our series was 640/mm^3^. The CD4 T cell count, which was 200 elements/mm^3^ in the series by Roblot et al. [14] but was not investigated in our investigation, appears to be less linked with PCP than in HIV patients [6]. In addition, the amount of C-reactive protein is greater, measuring 120 mg/L (60-210 mg/L) as opposed to 48 mg/L (17-128 mg/L) in HIV infection [10-14].

It might be challenging to make a positive PCP diagnosis in people who do not have HIV. In comparison to HIV infection, the pneumocystis burden in these patients is reduced [1].

Gomori-Grocott staining or immunofluorescence are used to detect the microorganism in induced sputum or BAL, but due to decreasing sensitivity and low fungal burden, new diagnostic tools such as quantitative PCR (qPCR) and b-D-glucan assay have been developed [14].

The sensitivity of polymerase chain reaction (PCR) in BAL (84%) and induced sputum (almost 100%) is outstanding, with specificities calculated at 90% and 83%, respectively. As a result, PCR has a good negative predictive value; however, as of 2015, it is not officially possible to distinguish between colonization and infection [15-17].

The measurement of serum levels of b-D-glucan, a component of the fungal wall and an indirect marker of fungal infection, can be used as an alternative to the diagnosis of pneumocystis. However, this test lacks pneumocystis specificity and is unable to identify some fungi, including Zygomycetes and *Blastomyces dermatitidis*.

Three types of patterns can be identified when examining the radiological data of pneumocystis in non-HIV patients: diffuse ground glass, inhomogeneously distributed, without connection with secondary lobules and with respect for the subpleural zone; condensations along the bronchovascular axes, with distortion and thickening of the interlobular septa; or ground glass with clear demarcation by the interlobular septa of the normal lung [5].

Trimethoprim/sulfamethoxazole (TMP/SMX) is used as first-line therapy for pulmonary pneumocystis at the following doses: TMP 15-20 mg/kg and SMX 75-100 mg/kg were administered in three to four doses daily for 21 days, while some authors contend that 14 days may be adequate in light of the lower fungus load in individuals without HIV who had their alveoli treated. Hypoxemic patients (PaO2: 70 mmHg) are given the following regimen: prednisone, 40 mg 2/day from day 1 to day 5, 40 mg/day from day 6 to day 10, and 20 mg/day from day 11 to day 21; adjuvant corticosteroid therapy should be combined [15,18].

The primary option, intravenous pentamidine, carries a high risk of significant side effects, including hypoglycemia, acute pancreatitis, and renal failure. Severe dosages of atovaquone or daily pentamidine sprays, which are less effective, should not be prescribed.

Clinicians now advise preventative medication for select patient groups due to the demonstrated effectiveness of primary prophylaxis in the treatment of AIDS [2].

**Figure 2:**
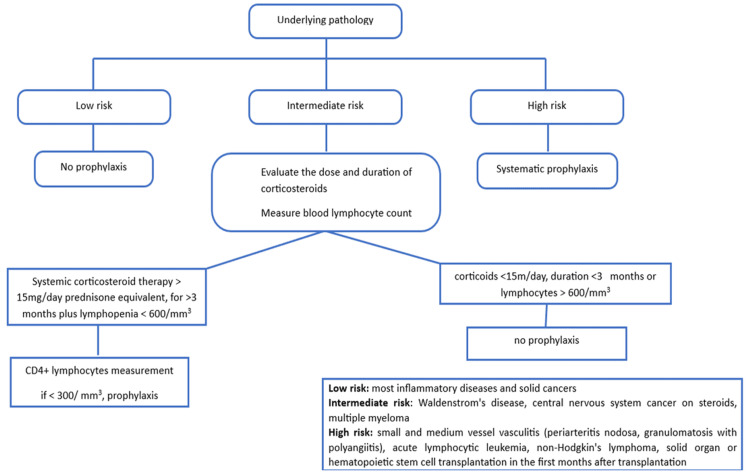
Decision support algorithm for pneumocystis prophylaxis in HIV-uninfected immunocompromised patients Adapted from [19,20] HIV: human immunodeficiency virus

We were able to estimate the incidence of pneumocystis for the main risk diseases in a single-center observational study by Fillâtre et al. [15], which included 154 cases of documented pneumocystis in non-HIV-infected patients. We did this by dividing the number of patients with pneumocystis for each disease by the total number of patients followed up for that disease during the study period. With the help of this research, we were able to rank the main immunosuppressive illnesses and their therapies in terms of the risk of pneumocystis (Figure 2) [19,20].

Steroids continue to be a PCP risk factor. In the trial by Yale et al. [21], doses of steroids equivalent to 16 mg of prednisone or higher provided for eight weeks were linked to a considerable chance of contracting pneumocystis. In the series by Roblot et al. [14], 57 patients were receiving long-term corticosteroid therapy at a mean daily dose of 46.3 mg. In one of our cases, extended corticosteroid medication had been administered for two years.

First-line prophylaxis is based on TMP/SMX and uses either 400 mg SMX/80 mg TMP (Bactrim simple®) once a day or 800 mg SMX/160 mg TMP (Bactrim forte®) three times a week. With a daily dose, one study hypothesized a marginally increased risk of adverse events [10,22], but this has not been verified [9].

In a side-by-side comparison study, 11% of patients receiving monthly pentamidine aerosols (300 mg) developed pneumocystis as opposed to none receiving TMP/SMX prophylaxis [23]. With incidences of pneumocystis assessed at 18.4 and 15.7 cases/100 patient-years, respectively, among the other oral prophylaxis that has been examined, dapsone (100 mg/day) and atovaquone (1,500 mg/day) are expected to have equal efficacy. However, atovaquone is more well tolerated.

In the series of Godeau et al., 13 out of 23 patients with systemic disorders had received secondary prophylaxis, suggesting that it is not necessarily essential. In patients receiving prophylaxis or not, no relapses were seen after 22 months [9]. In neither our series nor the study of Ninin et al. [13] were any relapses reported, while the series of Sepkowitz et al. [5] documented two cases out of 254 patients (Figure 2).

For reasons that are not fully understood but may include diagnosis at a more advanced stage (more rapid progression of lesions and more difficult diagnosis) and difficulties in correcting the immunodeficiency (more straightforward in the course of AIDS, since the advent of highly effective combined antiretroviral treatments), pulmonary pneumocystis remains serious in immunocompromised patients outside of HIV infection with mortality ranging from 20% to 60% [11,13]. In our study, no deaths were found, although severe respiratory distress was observed in 11 instances, four of which had benefited from noninvasive mechanical ventilation. There were 12 severe instances in the series by Ficko et al. [11], of which nine received mechanical ventilation and resulted in seven deaths; there were only two fatalities in the series by Battikh et al. [10].

We did not find any limitations in our study. Our patients' data were accessible.

## Conclusions

Pneumocystis is a serious illness with a rapid onset and a high fatality rate in patients who are not affected by HIV. TMP/SMX is still the first line of treatment, and corticosteroid therapy should only be used in hypoxemic patients. The course of treatment lasts for 21 days. It is necessary to include non-HIV immunosuppressed individuals in current education initiatives for HIV patients. Numerous premature deaths could be avoided if the same success is attained. Prophylaxis recommendations ought to be based on a number of factors, including the underlying illness, immunosuppressive medications taken, and total lymphocyte count.

## References

[1] Rouyer M, Stoclin A, Blanc FX (2015). [Pneumocystis pneumonia in HIV-negative adults]. Rev Mal Respir.

[2] Adler D, Chenivesse C, Similowski T, Soccal PM (2008). [Pneumocystis pneumonia in patients with immunosuppression other than HIV infection]. Rev Med Suisse.

[3] Burke BA, Good RA (1973). Pneumocystis carinii infection. Medicine (Baltimore).

[4] Roblot F, Godet C, Kauffmann C, Tattevin P, Boutoille D, Besnier JM, Hauet T (2009). Current predisposing factors for Pneumocystis pneumonia in immunocompromised HIV-negative patients. J Mycol Med.

[5] Sepkowitz KA, Brown AE, Telzak EE, Gottlieb S, Armstrong D (1992). Pneumocystis carinii pneumonia among patients without AIDS at a cancer hospital. JAMA.

[6] Toper C, Rivaud E, Daniel C (2011). [Pneumocystis jirovecii pneumonia in non-HIV infected patients: a study of 41 cases]. Rev Pneumol Clin.

[7] Catherinot E, Lanternier F, Bougnoux ME, Lecuit M, Couderc LJ, Lortholary O (2010). Pneumocystis jirovecii pneumonia. Infect Dis Clin North Am.

[8] Nevez G, Raccurt C, Jounieaux V, Dei-Cas E, Mazars E (1999). Pneumocystosis versus pulmonary Pneumocystis carinii colonization in HIV-negative and HIV-positive patients. AIDS.

[9] Tasaka S, Tokuda H (2012). Pneumocystis jirovecii pneumonia in non-HIV-infected patients in the era of novel immunosuppressive therapies. J Infect Chemother.

[10] Battikh R, M'Sadek F, Ben Abdelhafidh N (2007). Pneumocystis pneumonia in non HIV patients. Med Mal Infect.

[11] Ficko C, M'Rad MB, Suarez F, Catherinot E, Lortholary O, Guillevin L, Salmon D (2009). Pneumocystosis without HIV infection: a series of 28 cases (Article in French). Med Mal Infect.

[12] Roux A, Canet E, Valade S (2014). Pneumocystis jirovecii pneumonia in patients with or without AIDS, France. Emerg Infect Dis.

[13] Ninin E, Hamidou M, Germaud P, Morin O, Milpied N, Raffi F (1983). Pulmonary pneumocystosis in non-HIV patients: a retrospective study of 31 cases (Article in French). Presse Med.

[14] Roblot F, Godet C, Le Moal G (2002). Analysis of underlying diseases and prognosis factors associated with Pneumocystis carinii pneumonia in immunocompromised HIV-negative patients. Eur J Clin Microbiol Infect Dis.

[15] Fillâtre P, Revest M, Belaz S, Robert-Gangneux F, Zahar JR, Roblot F, Tattevin P (2016). [Pneumocystosis in non-HIV-infected immunocompromised patients]. Rev Med Interne.

[16] Olsson M, Strålin K, Holmberg H (2001). Clinical significance of nested polymerase chain reaction and immunofluorescence for detection of Pneumocystis carinii pneumonia. Clin Microbiol Infect.

[17] Tasaka S, Tokuda H, Sakai F (2010). Comparison of clinical and radiological features of pneumocystis pneumonia between malignancy cases and acquired immunodeficiency syndrome cases: a multicenter study. Intern Med.

[18] Moon SM, Kim T, Sung H (2011). Outcomes of moderate-to-severe Pneumocystis pneumonia treated with adjunctive steroid in non-HIV-infected patients. Antimicrob Agents Chemother.

[19] Fillatre P, Decaux O, Jouneau S (2014). Incidence of Pneumocystis jiroveci pneumonia among groups at risk in HIV-negative patients. Am J Med.

[20] Sowden E, Carmichael AJ (2004). Autoimmune inflammatory disorders, systemic corticosteroids and pneumocystis pneumonia: a strategy for prevention. BMC Infect Dis.

[21] El-Sadr WM, Luskin-Hawk R, Yurik TM (1999). A randomized trial of daily and thrice-weekly trimethoprim-sulfamethoxazole for the prevention of Pneumocystis carinii pneumonia in human immunodeficiency virus-infected persons. Terry Beirn Community Programs for Clinical Research on AIDS (CPCRA). Clin Infect Dis.

[22] Thomas CF Jr, Limper AH (2007). Current insights into the biology and pathogenesis of Pneumocystis pneumonia. Nat Rev Microbiol.

[23] Peron N, Roux A, Azoulay E (2015). Clinical and para-clinical aspects and prognostic factors of pneumocystosis in non-HIV subjects: study of 321 patients (Article in French). Rev Mal Respir.

